# Associations of Serum Liver Function Markers With Brain Structure, Function, and Perfusion in Healthy Young Adults

**DOI:** 10.3389/fneur.2021.606094

**Published:** 2021-02-25

**Authors:** Jingyao Chen, Siyu Liu, Chunli Wang, Cun Zhang, Huanhuan Cai, Min Zhang, Li Si, Shujun Zhang, Yuanhong Xu, Jiajia Zhu, Yongqiang Yu

**Affiliations:** ^1^Department of Radiology, The First Affiliated Hospital of Anhui Medical University, Hefei, China; ^2^Department of Clinical Laboratory, The First Affiliated Hospital of Anhui Medical University, Hefei, China; ^3^Medical Imaging Center, The First Affiliated Hospital of Anhui University of Chinese Medicine, Hefei, China

**Keywords:** magnetic resonance imaging, liver function, gray matter volume, regional homogeneity, cerebral blood flow, working memory

## Abstract

**Background:** Previous neuroimaging studies have demonstrated brain abnormalities in patients with hepatic diseases. However, the identified liver–brain associations are largely limited to disease-affected populations, and the nature and extent of such relations in healthy subjects remain unclear. We hypothesized that serum liver function markers within a normal level would affect brain properties.

**Method:** One hundred fifty-seven healthy young adults underwent structural, resting-state functional, and arterial spin labeling MRI scans. Gray matter volume (GMV), regional homogeneity (ReHo), and cerebral blood flow (CBF) analyses were performed to assess brain structure, function, and perfusion, respectively. Peripheral venous blood samples were collected to measure serum liver function markers. Correlation analyses were conducted to test potential associations between liver function markers and brain imaging parameters.

**Results:** First, serum proteins showed relations to brain structure characterized by higher albumin associated with increased GMV in the parahippocampal gyrus and amygdala and lower globulin and a higher albumin/globulin ratio with increased GMV in the olfactory cortex and parahippocampal gyrus. Second, serum bilirubin was linked to brain function characterized by higher bilirubin associated with increased ReHo in the precuneus, middle cingulate gyrus, inferior parietal lobule, and supramarginal gyrus and decreased ReHo in the caudate nucleus. Third, serum alanine transaminase (ALT) was related to brain perfusion characterized by higher ALT associated with increased CBF in the superior frontal gyrus and decreased CBF in the middle occipital gyrus, angular gyrus, precuneus, and middle temporal gyrus. More importantly, we found that CBF in the superior frontal gyrus was a significant mediator of the association between serum ALT level and working memory performance.

**Conclusion:** These findings may not only expand existing knowledge about the relationship between the liver and the brain but also have clinical implications for studying brain impairments secondary to liver diseases as well as providing potential neural targets for their diagnosis and treatment.

## Introduction

The liver acts as a major biochemical factory that carries out a number of synthetic and degradative processes. It is widely accepted that the liver plays a pivotal role in the metabolism, including glycogen storage, red blood cell decomposition, plasma protein synthesis, hormone production, and detoxification (1). Recently, emerging evidence supports a regulatory role of the central nervous system on liver function. Anatomically, the liver is innervated by sympathetic and parasympathetic fibers, such that the liver and the brain can interact in a bidirectional manner via autonomic nervous liver innervation (2). Potential communication pathways between the liver and the brain involve the neural, hormonal, and immune-mediated pathways (3, 4). Accordingly, it is logical to assume that liver diseases are by no means purely liver-centered diseases but, rather, frequently affect the brain through these potential pathways. For example, cognitive impairment is prevalent in patients with primary biliary cirrhosis (5). Hepatic encephalopathy in patients with liver failure manifests as abnormal behavior and compromised cognition (6). Inflammatory liver injury was found to be associated with changes in cerebral neurotransmission that result in sickness behaviors (7). A full explanation of these abnormal cognitive and behavioral phenomena observed in liver diseases might benefit from a better understanding of the brain–liver axis.

Advances in magnetic resonance imaging (MRI) techniques have provided a safe, non-invasive, and easily repeated neuroimaging avenue to explore the human brain *in vivo* (8, 9). Voxel-based morphometry (VBM) analysis of structural MRI, regional homogeneity (ReHo) analysis of resting-state functional MRI, and cerebral blood flow (CBF) analysis of arterial spin labeling (ASL) MRI allow for the measurement of brain structure, function, and perfusion, respectively (10–12). Using these approaches, pilot studies have demonstrated that patients with hepatic diseases show abnormalities in brain structure, function, and perfusion in widespread cortical, subcortical, and cerebellar areas (1, 13–19). However, these prior studies have been largely limited to examining the liver–brain associations in disease-affected populations and place less emphasis on investigating the nature and extent of such relations in healthy subjects.

Liver function can be assessed by peripheral blood levels of biochemical markers including albumin (ALB), globulin (GLO), ALB-to-GLO ratio (A/G ratio), bilirubin, alanine transaminase (ALT), and aspartate aminotransferase (AST). Specifically, ALB, GLO, and A/G ratio reflect the synthetic function of the liver (1). ALB is a plasma protein exclusively synthesized by the liver, and its reduction usually indicates liver disease (20). GLO is produced by the immune system and is thought to be implicated in damage response and related to the severity of liver inflammation (21). The A/G ratio takes both ALB and GLO into account, representing a sensitive index capable of detecting abnormalities of serum proteins (22). ALT and AST are enzymes that are engaged in the transfer of amino groups of alanine and aspartate to ketoglutaric acid. They are commonly used in clinical practice to measure hepatocyte injury (23) and serve as surrogate biomarkers of liver metabolic functioning (24). Bilirubin comes from the breakdown of senescent red blood cells. In the circulatory system, it predominantly circulates in its unconjugated form, termed indirect bilirubin (IBIL). When the IBIL binds to ALB, it turns into conjugated bilirubin, termed direct bilirubin (DBIL). Bilirubin plays a key role in the decomposition and absorption of lipids. Elevated bilirubin might suggest hepatocellular dysfunction or cholestasis (25–27).

In this study, we sought to explore the liver–brain associations in a large sample of healthy young adults. To this end, multiple serum biochemical markers (ALB, GLO, A/G ratio, bilirubin, ALT, and AST) were used to evaluate liver function; brain structure, function, and perfusion were measured by applying VBM, ReHo, and CBF analyses to multimodal MRI data. We hypothesized that serum liver function markers within a normal level would affect brain properties; moreover, there would be specificity of these effects in the way disparate serum liver function markers would be linked to distinct brain features in different regions.

## Materials and Methods

### Participants

A total of 157 healthy young adults were recruited by advertisement. All participants met the inclusion criteria of being Chinese Han, right-handed, and within a restricted age range of 18–30 years. Exclusion criteria included neuropsychiatric or severe somatic disorder, a history of alcohol or drug abuse, smoking regularly, current medication (e.g., antibiotics or sedative hypnotics) within a month, pregnancy, MRI contraindications, and a family history of psychiatric illness among first-degree relatives. The MINI-International Neuropsychiatric Interview (M.I.N.I.) and Alcohol Use Disorders Identification Test (AUDIT) were used in the process of excluding participants. This study was approved by the ethics committee of the First Affiliated Hospital of Anhui Medical University. Written informed consent was obtained from all participants after they had been given a complete description of the study. Demographic data of the sample are listed in Table 1.

**Table 1:** Demographic, cognitive, and serum liver function characteristics of 157 healthy participants.

**Characteristics**	**Mean ± SD**	**Range**
Gender (female/male)	77/80	–
Age (years)	22.3 ± 2.4	18–28
Education (years)	15.8 ± 1.9	12–20
FD (mm)	0.12 ± 0.05	0.04–0.40
TIV (cm3)	1,483.4 ± 132.7	1,102.2–1,789.9
ALB (g/L)	49.7 ± 2.5	43.6–56.9
GLO (g/L)	25.9 ± 2.9	20.6–35.7
A/G ratio	1.9 ± 0.3	1.3–2.6
TBIL (μmol/L)	14.9 ± 5.2	6.7–33.7
DBIL (μmol/L)	5.0 ± 2.2	1.1–12.1
IBIL (μmol/L)	9.9 ± 3.2	4.9–21.8
ALT (U/L)	18.3 ± 12.4	5–89
AST (U/L)	17.3 ± 6.4	9–53
3-back task performance		
Accuracy(%)	72.1 ± 15.7	15–98.3
Reaction time (ms)	768.9 ± 175.2	230.2–1,179.9

*SD, standard deviation; FD, frame-wise displacement; TIV, total intracranial volume; ALB, albumin; GLO, globulin; A/G, albumin/globulin; TBIL, total bilirubin; DBIL, direct bilirubin; IBIL, indirect bilirubin; ALT, alanine transaminase; AST, aspartate aminotransferase*.

### Blood Sampling and Measurement of Serum Liver Function Markers

After an overnight fasting period, peripheral venous blood samples (2 mL) were collected from all the participants in the morning. Samples were centrifuged to separate the serum at 3,000 rpm for 10 min at room temperature, and fresh serum was used immediately for the analysis of liver function markers including ALB, GLO, A/G ratio, total bilirubin (TBIL), DBIL, IBIL, ALT, and AST. The estimation of liver function markers was carried out in an automated clinical auto analyzer (Roche Cobas 8000).

### MRI Data Acquisition

MRI scans were obtained using a 3.0-Tesla MR system (Discovery MR750w, General Electric, Milwaukee, WI, USA) with a 24-channel head coil. Earplugs were used to reduce scanner noise, and tight but comfortable foam padding was used to minimize head motion. High-resolution 3D T1-weighted structural images were acquired by employing a brain volume (BRAVO) sequence with the following parameters: repetition time (TR) = 8.5 ms; echo time (TE) = 3.2 ms; inversion time (TI) = 450 ms; flip angle (FA) = 12°; field of view (FOV) = 256 × 256 mm; matrix size = 256 × 256; slice thickness = 1 mm, no gap; 188 sagittal slices. Resting-state blood oxygen level–dependent (BOLD) fMRI data were acquired using a gradient-echo single-shot echo planar imaging (GRE-SS-EPI) sequence with the following parameters: TR = 2,000 ms; TE = 30 ms; FA= 90°; FOV = 220 × 220 mm; matrix size = 64 × 64; slice thickness = 3 mm, slice gap = 1 mm; 35 interleaved axial slices; 185 volumes. The resting-state perfusion imaging was performed using a pseudo-continuous ASL sequence with a 3D fast spin-echo acquisition and background suppression (TR = 5,070 ms; TE = 11.5 ms; post-label delay = 2,025 ms; spiral in readout of eight arms with 512 sample points; FA = 111°; FOV = 240 × 240 mm; reconstruction matrix = 128 × 128; slice thickness = 3 mm, no gap; 50 axial slices; number of excitation = 3). The label and control whole-brain image volumes required eight TRs, respectively. A total of three pairs of label and control volumes were acquired. All images were visually inspected to ensure that only images without visible artifacts were included in subsequent analyses.

### Gray Matter Volume Analysis

VBM analysis was performed using the CAT12 toolbox (http://www.neuro.uni-jena.de/cat) implemented in the Statistical Parametric Mapping software (SPM12, *http://www.fil.ion.ucl.ac.uk/spm*). First, all the structural T1-weighted images were corrected for bias-field inhomogeneities. Second, these images were segmented into gray matter, white matter, and cerebrospinal fluid density maps using the “new-segment” approach (28). Third, a diffeomorphic anatomical registration through the exponentiated Lie algebra (DARTEL) technique was used to generate a custom, study-specific template (29). Fourth, each participant's gray matter density image was warped to the customized template; then the resultant images were affine registered to the Montreal Neurological Institute (MNI) space and resampled to a voxel size of 1.5 × 1.5 × 1.5 mm. Fifth, the modulation was applied by multiplying the transformed gray matter density maps with the nonlinear components of Jacobian determinants, which resulted in the normalized gray matter volume (GMV) maps representing the local native-space GMV after correcting the confounding effect of variance induced by individual whole-brain size. An automated anatomical labeling (AAL) template was employed to segment the cerebrum into 90 (45 for each hemisphere) cortical and subcortical regions of interest (ROIs) (30). Mean GMV within each ROI was extracted for subsequent ROI-based analysis.

### Regional Homogeneity Analysis

Resting-state BOLD data were preprocessed using the SPM12 and Data Processing & Analysis for Brain Imaging (DPABI, *http://rfmri.org/dpabi*) (31). The first 10 volumes for each participant were discarded to allow the signal to reach equilibrium and the participants to adapt to the scanning noise. The remaining volumes were corrected for the acquisition time delay between slices. Then, realignment was performed to correct the motion between time points. Head motion parameters were computed by estimating the translation in each direction and the angular rotation on each axis for each volume. All participants' BOLD data were within the defined motion thresholds (i.e., translational or rotational motion parameters less than 2 mm or 2°). We also calculated frame-wise displacement (FD), which indexes the volume-to-volume changes in head position. Several nuisance covariates (the linear drift, the estimated motion parameters based on the Friston-24 model, the spike volumes with FD > 0.5, the white matter signal, and the cerebrospinal fluid signal) were regressed out from the data. The datasets were then band-pass-filtered using a frequency range of 0.01–0.1 Hz. In the normalization step, individual structural images were firstly co-registered with the mean functional image; then the transformed structural images were segmented and normalized to the MNI space using a high-level nonlinear warping algorithm, that is, the DARTEL technique (29). Finally, each filtered functional volume was spatially normalized to MNI space using the deformation parameters estimated during the above step and resampled into a 3 mm cubic voxel.

The ReHo calculation procedure was the same as that reported in previous studies (11). ReHo can be used to measure the degree of regional neural activity coherence. In short, it was calculated as Kendall's coefficient of concordance (or Kendall's W) of the time course of a given voxel with those of its nearest neighbors (26 voxels). For the purpose of standardization, the ReHo value of each voxel was divided by the global mean ReHo value. An AAL template was employed to segment the cerebrum into 90 (45 for each hemisphere) cortical and subcortical ROIs (30). Mean ReHo within each ROI was extracted for subsequent ROI-based analysis.

### Cerebral Blood Flow Analysis

Three ASL difference images were calculated by subtracting the label images from the control images and then averaged. Next, CBF was quantified by applying a single-compartment model (12) to the mean ASL difference and proton density–weighted reference images (32–34). SPM12 software was used to normalize the CBF images into the MNI space using the following steps: (1) individual structural images were firstly co-registered with the CBF images; (2) the transformed structural images were segmented and normalized to the MNI space; and (3) the CBF image of each subject was written into the MNI space using the deformation parameter derived from the prior step and was resliced into a 2 mm cubic voxel. For the purpose of standardization, the CBF value of each voxel was divided by the global mean CBF value. An AAL template was utilized to segment the cerebrum into 90 (45 for each hemisphere) cortical and subcortical ROIs (30). Mean CBF within each ROI was extracted for subsequent ROI-based analysis.

### Working Memory Assessment

The letter 3-back task was conducted on a computer to assess working memory (35) using E-Prime 2.0 (*http://www.pstnet.com/eprime.cfm*). During the task, each participant viewed a series of letters that were presented sequentially, and the presentation time of each letter stimulus was 200 ms with an inter-stimulus interval of 1,800 ms. Participants were instructed to press a button on the right with their middle finger if the letter that appeared on the screen was identical to the one presented three letters earlier and otherwise to press a button on the left with their index finger. The task consisted of 60 trials. Before the formal test, participants were verbally instructed and had a practice test to ensure that they understood the task. The accuracy and mean reaction time of correct responses were used as the indices of working memory performance.

### Statistical Analysis

The SPSS 23.0 software package (SPSS, Chicago, Ill) was used to perform the following statistical analyses. Partial correlation analyses were performed to test the associations between serum liver function markers (ALB, GLO, A/G ratio, TBIL, DBIL, IBIL, ALT, and AST) and ROI-based brain imaging parameters (GMV, ReHo, and CBF). For CBF analyses, age and gender were included as nuisance covariates, with total intracranial volume (TIV) and FD as additional covariates for GMV and ReHo analyses respectively. For brain imaging parameters showing correlations with serum liver function markers, we further examined their associations with working memory performance (3-back accuracy and mean reaction time) using partial correlations. The significance threshold was set at two-tailed *p* < 0.01 to balance the risk for type I and type II errors. No corrections for multiple testing were conducted, as our objective for this exploratory research was to generate some hypotheses for further testing and confirmation in a larger sample.

To test whether the association between variables was mediated by other variables, mediation analysis was performed using the PROCESS macro (http://www.processmacro.org/) developed by Hayes (36). PROCESS uses an ordinary least squares path analytic framework to estimate direct and indirect mediation effects. In the mediation analysis model, all paths were reported as unstandardized ordinary least squares regression coefficients, namely, total effect of X on Y (c) = indirect effect of X on Y through M (a × b) + direct effect of X on Y (c'). The significance analysis was based on 5,000 bootstrap realizations, and the significance of indirect effects was assessed by bootstrap 95% confidence interval (CI). In the PROCESS analysis, a significant indirect effect is indicated when the bootstrap 95% CI does not include zero. In this study, only variables that showed a significant correlation with others were considered independent (serum liver function markers), dependent (working memory performance), or mediating (brain imaging parameters) variables in the mediation analysis.

## Results

### Correlations Between Serum Proteins and GMV

**Figure 1:**
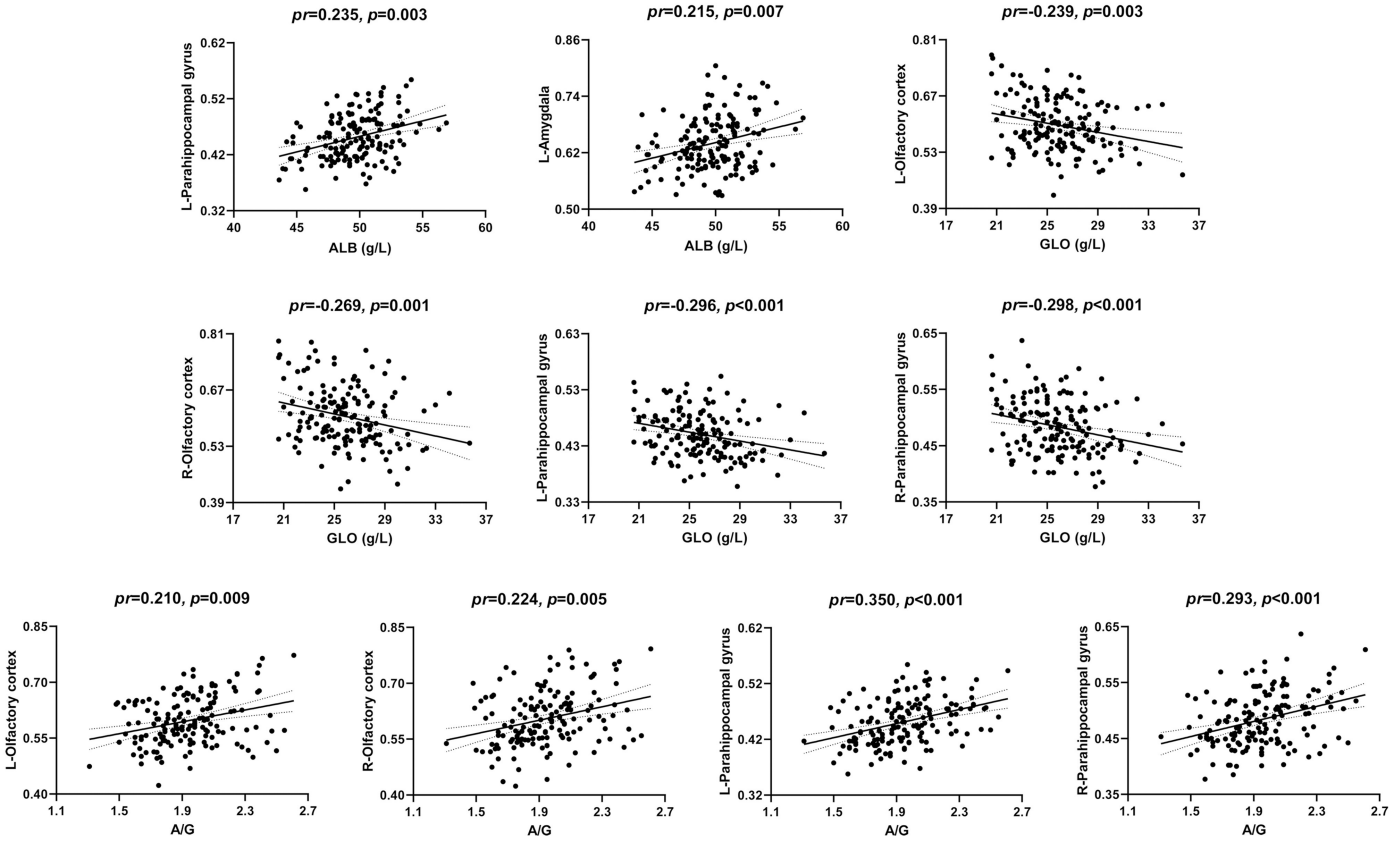
Scatter plots of the correlations between serum proteins and GMV. Abbreviations: GMV, gray matter volume; ALB, albumin; GLO, globulin; A/G, albumin/globulin; *pr*, partial correlation coefficient; L, left; R, right.

**Figure 2:**
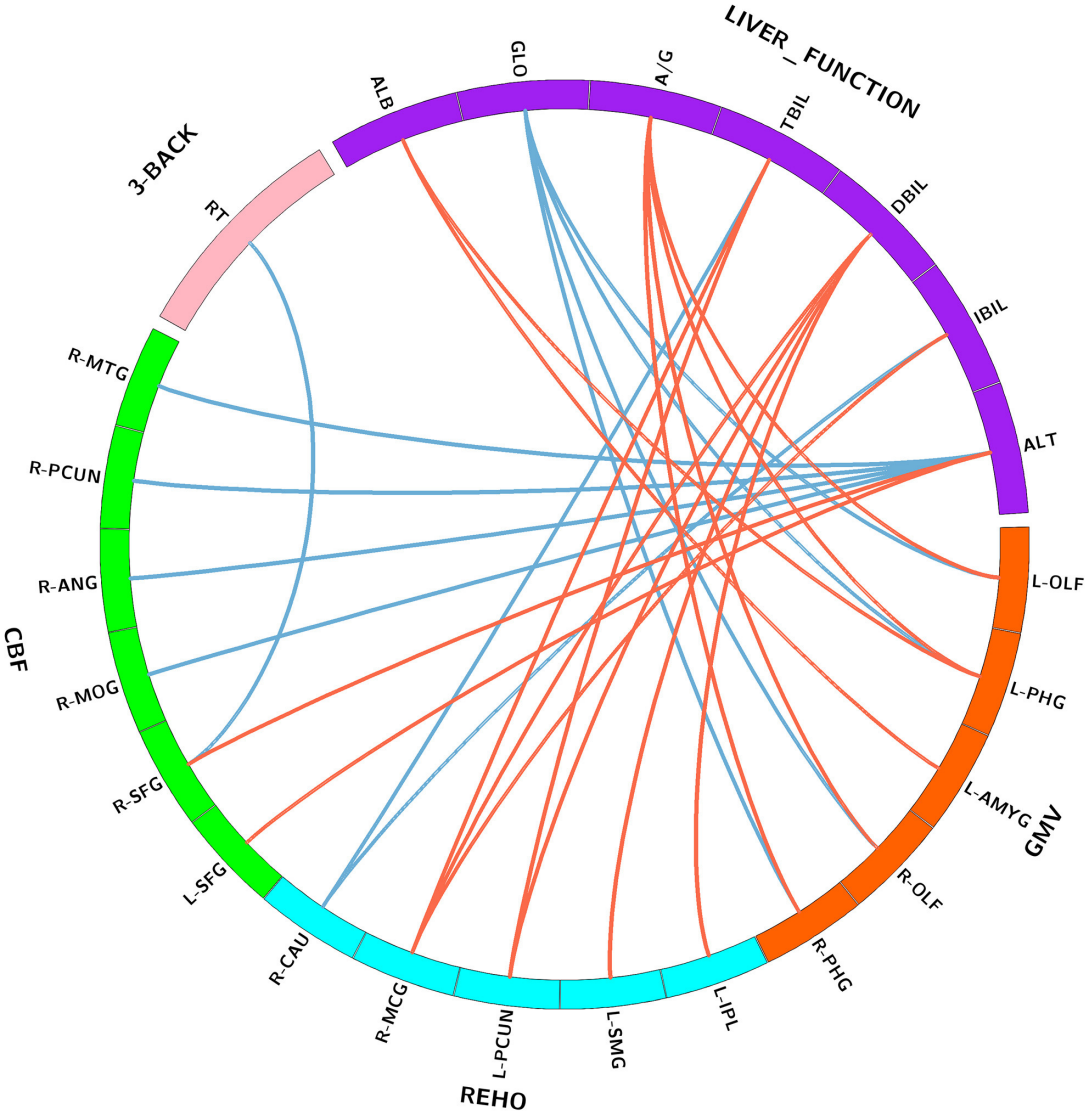
Correlations between serum liver function markers, brain imaging parameters, and working memory performance. Purple block represents serum liver function markers; orange block represents GMV; blue block represents ReHo; green block represents CBF; and pink block represents 3-back task performance. Red lines indicate positive correlations, and blue lines indicate negative correlations. Abbreviations: GMV, gray matter volume; ReHo, regional homogeneity; CBF, cerebral blood flow; ALB, albumin; GLO, globulin; A/G, albumin/globulin; TBIL, total bilirubin; DBIL, direct bilirubin; IBIL, indirect bilirubin; ALT, alanine transaminase; OLF, olfactory cortex; PHG, parahippocampal; AMYG, amygdala; IPL, inferior parietal lobule; SMG, supramarginal gyrus; PCUN, precuneus; MCG, middle cingulate gyrus; CAU, caudate nucleus; SFG, superior frontal gyrus; MOG, middle occipital gyrus; ANG, angular gyrus; MTG, middle temporal gyrus; RT, reaction time; L, left; R, right.

**Figure 3:**
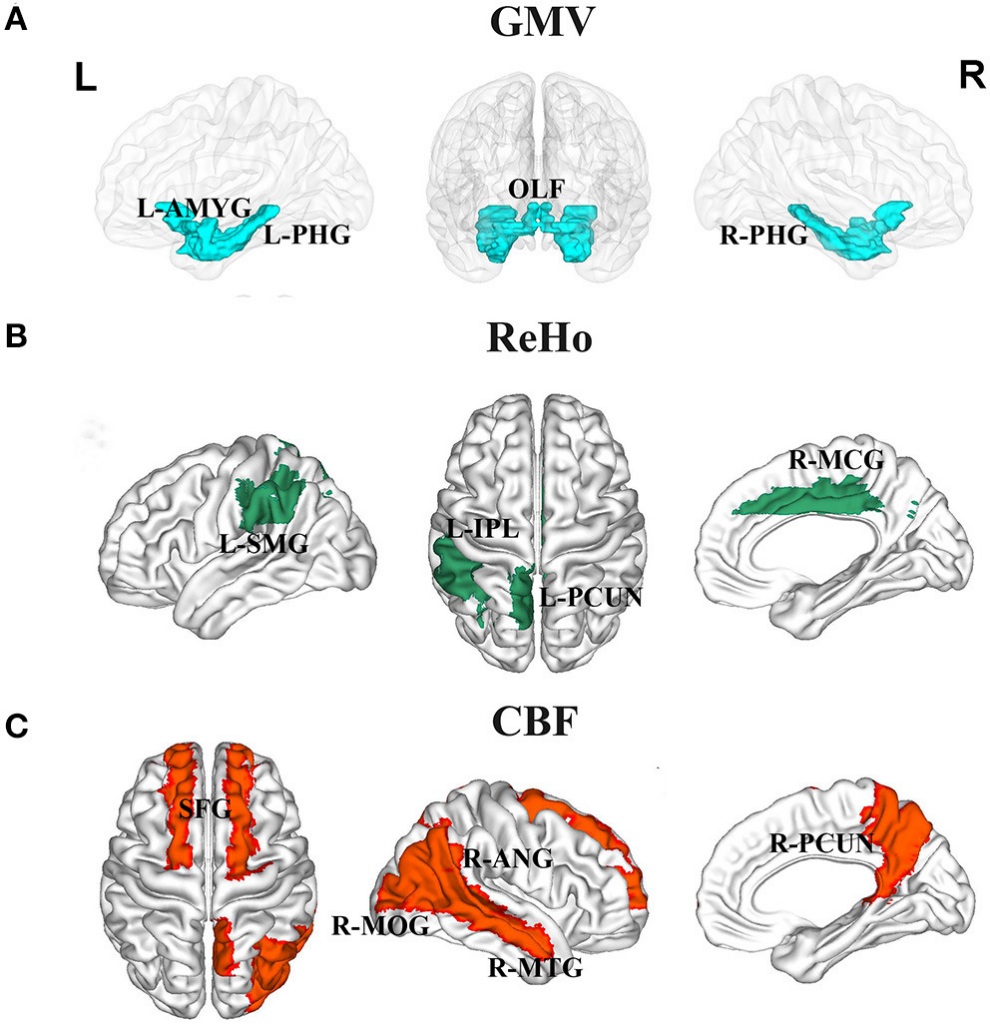
Brain regions associated with serum liver function markers. **(A)** GMV in the left amygdala, bilateral olfactory cortex, and bilateral parahippocampal gyrus were related to serum protein levels. **(B)** ReHo in the left inferior parietal lobule, left supramarginal gyrus, left precuneus, and right middle cingulate gyrus were related to serum bilirubin levels. **(C)** CBF in the bilateral superior frontal gyrus, right middle occipital gyrus, right angular gyrus, right precuneus, and right middle temporal gyrus were related to serum ALT level. Abbreviations: GMV, gray matter volume; ReHo, regional homogeneity; CBF, cerebral blood flow; ALT, alanine transaminase; OLF, olfactory cortex; PHG, parahippocampal; AMYG, amygdala; IPL, inferior parietal lobule; SMG, supramarginal gyrus; PCUN, precuneus; MCG, middle cingulate gyrus; SFG, superior frontal gyrus; MOG, middle occipital gyrus; ANG, angular gyrus; MTG, middle temporal gyrus; L, left; R, right.

The correlations between serum protein levels and GMV are illustrated in Figures 1, 2. After adjustment for age, gender, and TIV, we found significant positive correlations between ALB and GMV in the left parahippocampal gyrus (*pr* = 0.235, *p* = 0.003) and left amygdala (*pr* = 0.215, *p* = 0.007); negative correlations between GLO and GMV in the bilateral olfactory cortex (left: *pr* = −0.239, *p* = 0.003; right: *pr* = −0.269, *p* = 0.001) and bilateral parahippocampal gyrus (left: *pr* = −0.296, *p* < 0.001; right: *pr* = −0.298, *p* < 0.001); and positive correlations between A/G ratio and GMV in the bilateral olfactory cortex (left: *pr* = 0.210, *p* = 0.009; right: *pr* = 0.224, *p* = 0.005) and bilateral parahippocampal gyrus (left: *pr* = 0.350, *p* < 0.001; right: *pr* = 0.293, *p* < 0.001). These brain regions associated with serum protein levels are shown in Figure 3A. However, there were no significant correlations of serum proteins with ReHo and CBF (*p* > 0.01).

### Correlations Between Serum Bilirubin and ReHo

**Figure 4:**
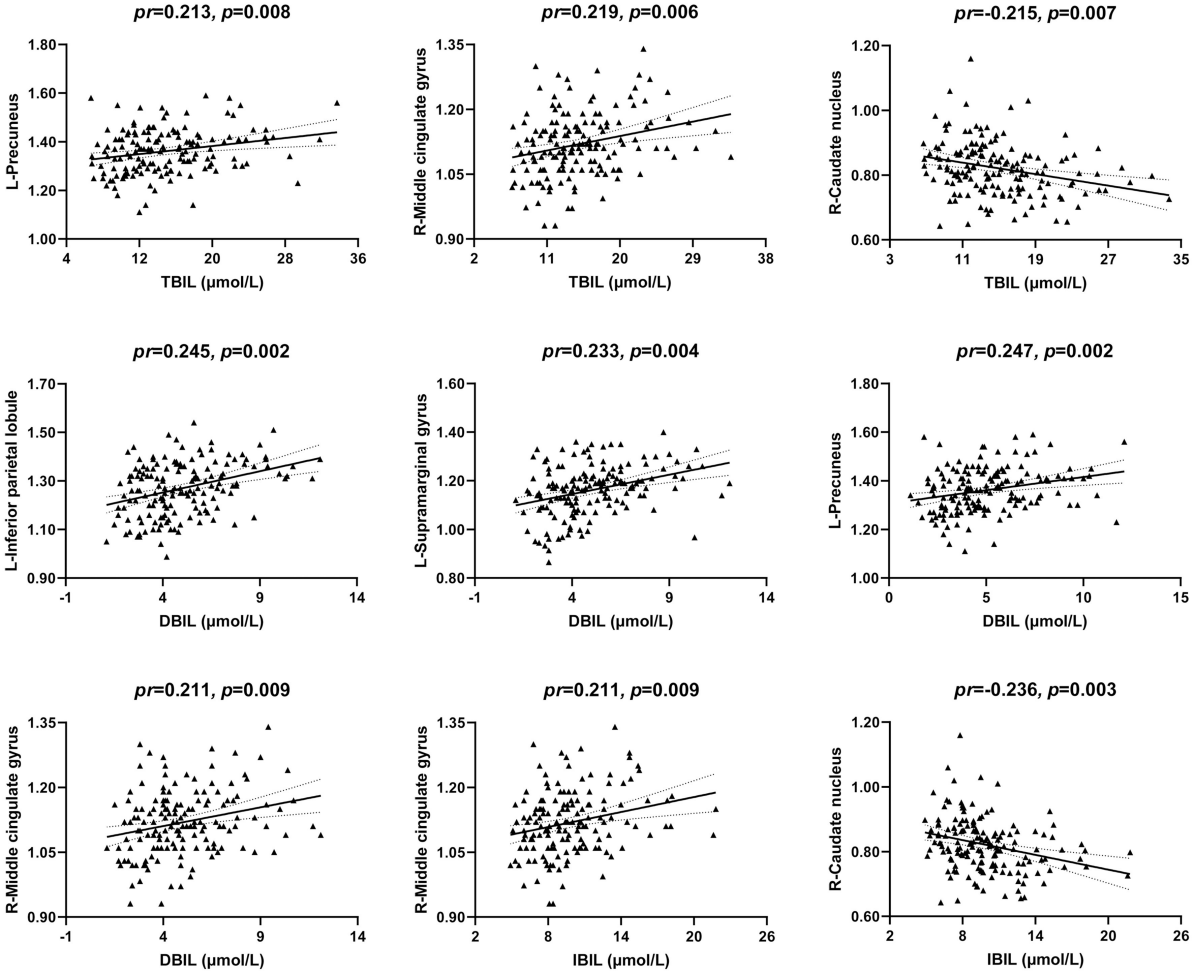
Scatter plots of the correlations between serum bilirubin and ReHo. Abbreviations: ReHo, regional homogeneity; TBIL, total bilirubin; DBIL, direct bilirubin; IBIL, indirect bilirubin; *pr*, partial correlation coefficient; L, left; R, right.

The correlations between serum bilirubin levels and ReHo are illustrated in Figures 4, 2. After controlling for age, gender, and FD, serum TBIL level exhibited significant positive correlations with ReHo in the left precuneus (*pr* = 0.213, *p* = 0.008) and right middle cingulate gyrus (MCG) (*pr* = 0.219, *p* = 0.006) and a negative correlation with ReHo in the right caudate nucleus (*pr* = −0.215, *p* = 0.007). There were significant positive correlations between DBIL and ReHo in the left inferior parietal lobule (IPL) (*pr* = 0.245, *p* = 0.002), left supramarginal gyrus (*pr* = 0.233, *p* = 0.004), left precuneus (*pr* = 0.247, *p* = 0.002), and right MCG (*pr* = 0.211, *p* = 0.009). In addition, IBIL level was positively correlated with ReHo in the right MCG (*pr* = 0.211, *p* = 0.009) and negatively associated with ReHo in the right caudate nucleus (*pr* = −0.236, *p* = 0.003). These brain regions associated with serum bilirubin levels, with the exception of the right caudate nucleus, are shown in Figure 3B. However, there were no significant correlations of serum bilirubin with GMV and CBF (*p* > 0.01).

### Correlations Between Serum ALT and CBF

**Figure 5:**
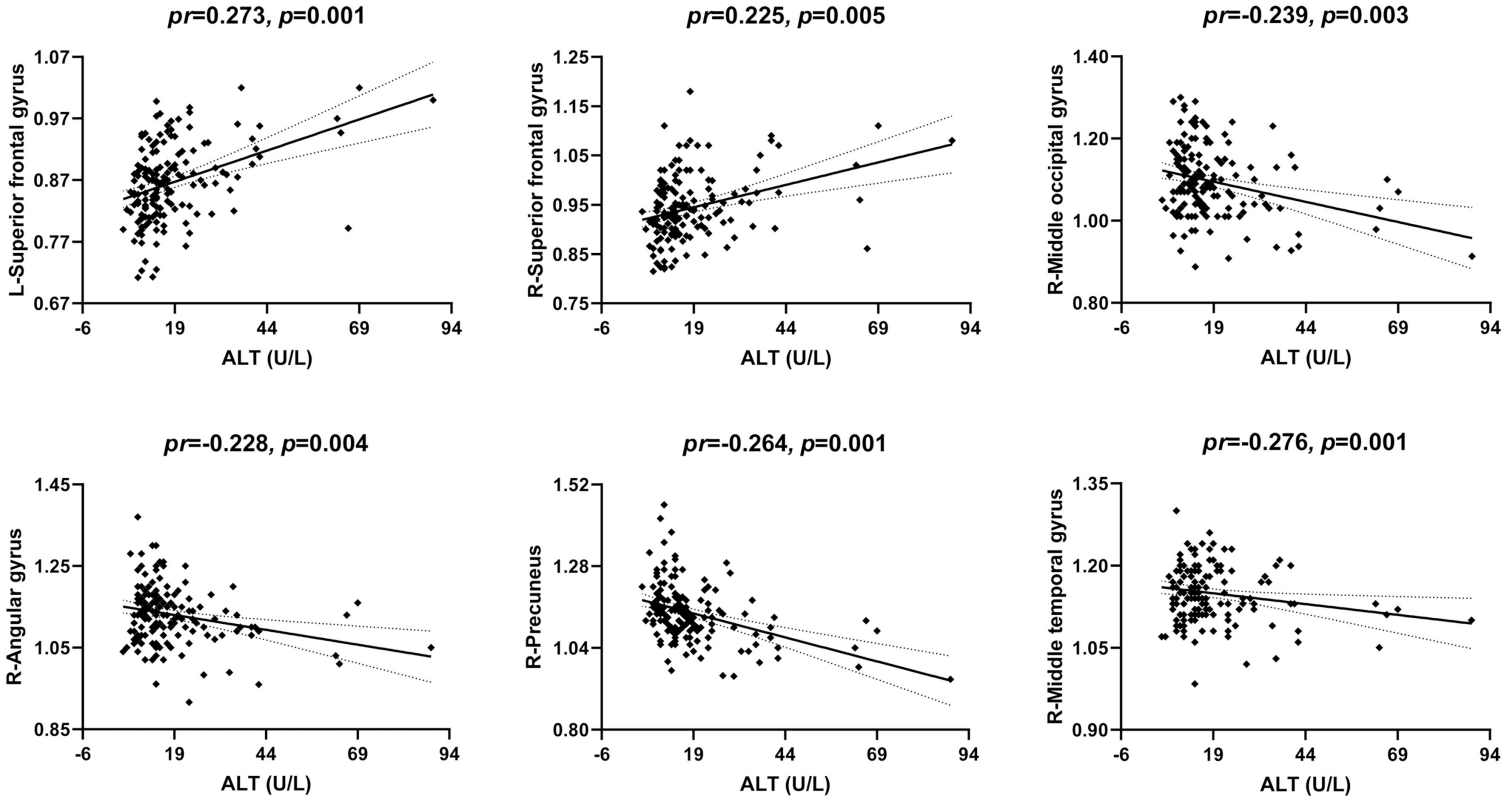
Scatter plots of the correlations between serum ALT and CBF. Abbreviations: ALT, alanine transaminase; CBF, cerebral blood flow; *pr*, partial correlation coefficient; L, left; R, right.

The correlations between serum ALT level and CBF are illustrated in Figures 5, 2. After accounting for age and gender, serum ALT level showed significant positive correlations with CBF in the bilateral superior frontal gyrus (SFG) (left: *pr* = 0.273, *p* = 0.001; right: *pr* = 0.225, *p* = 0.005) and negative correlations with CBF in the right middle occipital gyrus (MOG) (*pr* = −0.239, *p* = 0.003), right angular gyrus (*pr* = −0.228, *p* = 0.004), right precuneus (*pr* = −0.264, *p* = 0.001), and right middle temporal gyrus (MTG) (*pr* = −0.276, *p* = 0.001). These brain regions associated with serum ALT level are shown in Figure 3C. However, there were no significant correlations between serum AST and CBF (*p* > 0.01). There were also no significant correlations of serum ALT and AST with GMV and ReHo (*p* > 0.01).

### CBF Mediating the Relationship Between Serum ALT and Working Memory

**Figure 6:**
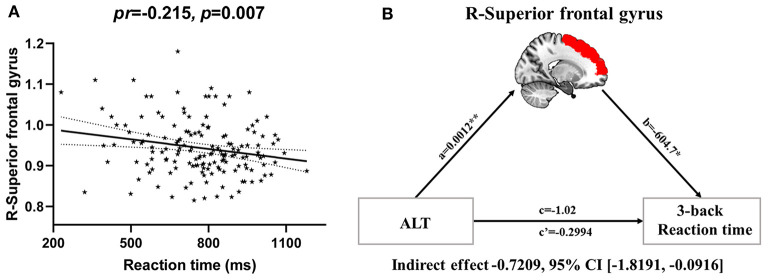
CBF mediating the relationship between serum ALT and working memory. **(A)** Scatter plot of the correlation between CBF in the right superior frontal gyrus and 3-back mean reaction time. **(B)** The mediation analysis between serum ALT level (X) and 3-back reaction time (Y), with CBF in the right superior frontal gyrus as the mediator (M). Path coefficients with *p* values (*p < 0.05 and **p < 0.01, respectively). Abbreviations: CBF, cerebral blood flow; ALT, alanine transaminase.

Partial correlation analyses revealed a significant negative correlation between CBF in the right SFG and 3-back mean reaction time (*pr* = −0.215, *p* = 0.007) (Figures 6A, 2). However, no significant correlation between brain imaging parameters and 3-back accuracy was present. Further, mediation analysis demonstrated that the relationship between serum ALT level and 3-back mean reaction time was significantly mediated by CBF in the right SFG (indirect effect = −0.7209, SE = 0.4134, 95% CI: −1.8191, −0.0916) after controlling for age and gender (Figure 6B).

### Sensitivity Analysis

To test the possible effects of extreme outliers on our results, we repeated the partial correlation analyses after excluding participants with serum liver function values greater than mean + 3 × standard deviation (SD) or smaller than mean – 3 × SD. As shown in Supplementary Tables S1–3, despite slight changes in *p* values, all of the aforementioned correlations were still significant after excluding the outliers (*p* < 0.05). Notably, we also included education as an additional nuisance covariate when assessing the correlations between serum liver function markers and MRI parameters. As shown in Supplementary Tables S4–6, our main results were preserved after additional adjustment for education (*p* < 0.01).

## Discussion

Using multimodal MRI approaches, we examined the associations between serum liver function markers and brain imaging parameters in a cohort of healthy young adults. Four main findings were observed in the present study. First, serum proteins showed relations to brain structure characterized by higher ALB associated with increased GMV in the parahippocampal gyrus and amygdala and lower GLO and higher A/G ratio with increased GMV in the olfactory cortex and parahippocampal gyrus. Second, serum bilirubin was linked to brain function characterized by higher bilirubin associated with increased ReHo in the precuneus, MCG, IPL, and supramarginal gyrus and decreased ReHo in the caudate nucleus. Third, serum ALT was related to brain perfusion characterized by higher ALT associated with increased CBF in the SFG and decreased CBF in the MOG, angular gyrus, precuneus, and MTG. Finally, higher CBF in the right SFG was correlated with shorter 3-back reaction time; more importantly, mediation analysis further revealed that CBF in the right SFG was a significant mediator of the association between serum ALT level and working memory performance.

ALB is the major protein component in blood, accounting for 50–60% of the total proteins (37). Its reduction has been associated with malnutrition, chronic infections, and chronic liver diseases (20, 38). There has been strong evidence for the association between serum ALB and cognitive function. For instance, low serum ALB was found to be independently associated with increased odds of cognitive impairment in the elderly population (39). There was also a positive correlation between ALB and cognitive performance in physiologically healthy participants (40). Conversely, GLO has been reported to increase during chronic inflammation, which has been linked to cognitive decline (3). Serum A/G ratio, which combines ALB and GLO, has been reported to positively relate to cognitive function (22, 41). Building on earlier research, it is reasonable to postulate that serum ALB and A/G ratio may serve as protective factors for cognition, while serum GLO may be a potential risk factor. This notion is also supported by our current observation that GMV in the limbic regions was positively correlated with ALB and A/G ratio yet inversely correlated with GLO. The parahippocampal gyrus is associated with many cognitive processes, including visuospatial processing and episodic memory (42). The amygdala is a multifaceted construct that contributes strongly to emotion and cognition (43–45). Structural and functional abnormalities in the olfactory cortex were detected in patients with cognitive deficits (46). Impaired olfaction was predictive of cognition decline in nondemented older adults (47). These reports are suggestive of a possible link between cognition and the olfactory cortex. Collectively, our results, coupled with these prior findings, may help further elucidate the relationships between serum proteins and cognitive function by identifying potential neural correlates.

We found that higher bilirubin was associated with increased ReHo in the MCG, precuneus, IPL, and supramarginal gyrus. Caruana et al. reported that the MCG contributed to complex motor behaviors and sensory modalities by using electrical stimulation (48). The precuneus, IPL, and supramarginal gyrus have been documented to involve visual information processing (49, 50). Previous work has revealed that bilirubin exhibits powerful antioxidative and anti-inflammatory effects (51, 52) and is thus considered a neuroprotective factor acting by scavenging superoxide during neurotransmission (27). These positive correlations between bilirubin and local neural activity in these brain regions may reflect protective effects of bilirubin on motor and sensory systems. By contrast, we observed a negative correlation between serum bilirubin and ReHo in the caudate nucleus. Ni and colleagues found increased ReHo in the caudate nucleus in cirrhotic patients (53). Our present findings, taken with those of Ni et al., highlight the critical role of the caudate nucleus in the brain–liver communication pathway in both healthy and clinical populations.

Our data showed that higher serum ALT was correlated with decreased CBF in the MOG, angular gyrus, precuneus, and MTG in healthy young adults. The MOG and MTG are involved in the processing of visual cues (54, 55), and the MOG and precuneus, in top-down visuospatial function (49). From the viewpoint of networks, the precuneus and angular gyrus are core regions of the default-mode network (DMN) (56). In healthy individuals, increased ALT was considered a biomarker of hepatic insulin resistance (57) and could be used to predict the development of type 2 diabetes (58). By using perfusion fMRI, a previous study demonstrated that patients with type 2 diabetes exhibited decreased CBF in the visual areas and DMN (59). On the basis of these findings, one may speculate that brain glucose hypometabolism might explain the associations between an increased level of ALT and hypoperfusion in the visual and DMN brain regions. In contrast, our analyses demonstrated that higher serum ALT was correlated with higher CBF in the SFG. The SFG is generally considered a hub region implicating cognitive control (60) and emotion regulation (61). While preliminary, this association appears to be compensatory.

Previous work has established that serum ALT level is associated with cognition in healthy and clinical populations (62–64). Besides, it is generally accepted that the SFG contributes to higher cognitive functions and particularly to working memory (65, 66). In line with these prior reports, our mediation analysis demonstrates that higher serum ALT leads to higher CBF in the SFG, which in turn results in shorter 3-back mean reaction time. This finding of a positive effect of serum ALT level on working memory performance via CBF in the SFG in healthy young adults not only may complement and extend previous literature on the relationships between liver function biomarkers and cognition but also may help shed light on the neural mechanism underlying such associations.

There are several limitations that should be mentioned in this study. First, causal relationships cannot be inferred from this cross-sectional design. Longitudinal studies with intervention targeted toward altering levels of liver function markers in patients with hepatic diseases are needed to establish the direction of causality. Second, given that this study population was selected from a group of educated volunteers with an age range of 18–30 years, the current findings might not be representative of the general population. Future investigations are warranted to further improve our understanding of the liver–brain relationship by enrolling a sample of subjects with broader age and educational ranges. Third, multiple testing corrections were not performed for the correlation analyses, because our results did not survive correction, likely due to the modest sample size and/or relatively small effect size of the liver–brain association. However, our analyses were exploratory in nature and important for future hypothesis generation. Therefore, we reported uncorrected *p* values, as Type II error control is equally important in exploratory research. Finally, although we established some associations between serum liver function markers and brain properties, the biological mechanisms underlying these associations remain unclear and need to be further determined in the future.

In conclusion, this is, to our knowledge, the first multimodal MRI study demonstrating associations of serum liver function markers with brain structure, function, and perfusion in a large cohort of healthy young adults. The observed relations of serum proteins with GMV, bilirubin with ReHo, and ALT with CBF may help to expand existing knowledge about the relationship between the liver and the brain by indicating the specificity of these effects in the way that disparate serum liver function markers are linked to distinct brain features in different regions. More broadly, these findings may have clinical implications for studying brain impairments secondary to liver diseases as well as providing potential neural targets for their diagnosis and treatment.

## Data Availability Statement

The datasets for this article are not publicly available to protect the patient's privacy. Requests to access the datasets should be directed to cjr.yuyongqiang@vip.163.com.

## Ethics Statement

The studies involving human participants were reviewed and approved by the ethics committee of The First Affiliated Hospital of Anhui Medical University. All participants provided written informed consent after they had been given a complete description of the study. The patients/participants provided their written informed consent to participate in this study.

## Author Contributions

JC, SL, and CW: methodology, data curation, software, and writing—original draft. CZ, HC, MZ, LS, SZ, and YX: data collection, visualization, and investigation. JZ: conceptualization, methodology, software, formal analysis, and writing—review and editing. YY: conceptualization, supervision, and writing—review and editing. All authors contributed to the article and approved the submitted version.

## Conflict of Interest

The authors declare that the research was conducted in the absence of any commercial or financial relationships that could be construed as a potential conflict of interest.
