# Supplementary material for: Associations of Serum Liver Function Markers With Brain Structure, Function, and Perfusion in Healthy Young Adults
Source: Front Neurol. 2021 Feb 25;12:606094. doi: 10.3389/fneur.2021.606094 (PMC7947675; doi:10.3389/fneur.2021.606094)
Supplement: Supplementary file 1 [file Data_Sheet_1.pdf]

Supplementary materials

Tables

Table S1. The correlations between serum proteins and GMV after excluding the outliers

Serum proteins	Brain regions	*pr*	*p*
ALB (g/L)	L-parahippocamal gyrus	0.235	0.003
ALB (g/L)	L-amygdala	0.215	0.007
GLO (g/L)	L-olfactory cortex	-0.180	0.026
GLO (g/L)	R-olfactory cortex	-0.238	0.003
GLO (g/L)	L-parahippocamal gyrus	-0.267	0.001
GLO (g/L)	R-parahippocamal gyrus	-0.269	0.001
A/G ratio	L-olfactory cortex	0.210	0.009
A/G ratio	R-olfactory cortex	0.224	0.005
A/G ratio	L-parahippocamal gyrus	0.350	< 0.001
A/G ratio	R-parahippocamal gyrus	0.293	< 0.001

Abbreviations: GMV, gray matter volume; ALB, albumin; GLO, globulin; A/G, albumin/globulin; *pr*, partial correlation coefficient; L, left; R, right.

Table S2. The correlations between serum bilirubin and ReHo after excluding the outliers

Serum bilirubin	Brain regions	*pr*	*p*
TBIL (μmol/L)	L-precuneus	0.174	0.032
TBIL (μmol/L)	R-middle cingulate gyrus	0.243	0.003
TBIL (μmol/L)	R-caudate nucleus	-0.213	0.008
DBIL (μmol/L)	L-inferior parietal lobule	0.245	0.002
DBIL (μmol/L)	L-supramarginal gyrus	0.262	0.001
DBIL (μmol/L)	L-precuneus	0.255	0.001
DBIL (μmol/L)	R-middle cingulate gyrus	0.242	0.003
IBIL (μmol/L)	R-middle cingulate gyrus	0.237	0.003
IBIL (μmol/L)	R-caudate nucleus	-0.239	0.003

Abbreviations: ReHo, regional homogeneity; TBIL, total bilirubin; DBIL, direct bilirubin; IBIL, indirect bilirubin; *pr*, partial correlation coefficient; L, left; R, right.

Table S3. The correlations between serum ALT and CBF after excluding the outliers

Serum ALT	Brain regions	*pr*	*p*
ALT (U/L)	L-superior frontal gyrus	0.212	0.009
ALT (U/L)	R-superior frontal gyrus	0.186	0.023
ALT (U/L)	R-middle occipital gyrus	-0.194	0.017
ALT (U/L)	R-angular gyrus	-0.221	0.007
ALT (U/L)	R-precuneus	-0.214	0.009
ALT (U/L)	R-middle temporal gyrus	-0.198	0.015

Abbreviations: ALT, alanine transaminase; CBF, cerebral blood flow; *pr*, partial correlation coefficient; L, left; R, right.

Table S4. The correlations between serum proteins and GMV after additional adjustment for education

Serum proteins	Brain regions	*pr*	*p*
ALB (g/L)	L-parahippocamal gyrus	0.235	0.003
ALB (g/L)	L-amygdala	0.214	0.008
GLO (g/L)	L-olfactory cortex	-0.248	0.002
GLO (g/L)	R-olfactory cortex	-0.282	< 0.001
GLO (g/L)	L-parahippocamal gyrus	-0.291	< 0.001
GLO (g/L)	R-parahippocamal gyrus	-0.301	< 0.001
A/G ratio	L-olfactory cortex	0.22	0.006
A/G ratio	R-olfactory cortex	0.237	0.003
A/G ratio	L-parahippocamal gyrus	0.344	< 0.001
A/G ratio	R-parahippocamal gyrus	0.296	< 0.001

Abbreviations: GMV, gray matter volume; ALB, albumin; GLO, globulin; A/G, albumin/globulin; *pr*, partial correlation coefficient; L, left; R, right.

Table S5. The correlations between serum bilirubin and ReHo after additional adjustment for education

Serum bilirubin	Brain regions	*pr*	*p*
TBIL (μmol/L)	L-precuneus	0.214	0.008
TBIL (μmol/L)	R-middle cingulate gyrus	0.219	0.007
TBIL (μmol/L)	R-caudate nucleus	-0.216	0.007
DBIL (μmol/L)	L-inferior parietal lobule	0.245	0.002
DBIL (μmol/L)	L-supramarginal gyrus	0.232	0.004
DBIL (μmol/L)	L-precuneus	0.246	0.002
DBIL (μmol/L)	R-middle cingulate gyrus	0.21	0.009
IBIL (μmol/L)	R-middle cingulate gyrus	0.212	0.009
IBIL (μmol/L)	R-caudate nucleus	-0.237	0.003

Abbreviations: ReHo, regional homogeneity; TBIL, total bilirubin; DBIL, direct bilirubin; IBIL, indirect bilirubin; *pr*, partial correlation coefficient; L, left; R, right.

Table S6. The correlations between serum ALT and CBF after additional adjustment for education

Serum ALT	Brain regions	*pr*	*p*
ALT (U/L)	L-superior frontal gyrus	0.272	0.001
ALT (U/L)	R-superior frontal gyrus	0.226	0.005
ALT (U/L)	R-middle occipital gyrus	-0.24	0.003
ALT (U/L)	R-angular gyrus	-0.228	0.005
ALT (U/L)	R-precuneus	-0.263	0.001
ALT (U/L)	R-middle temporal gyrus	-0.275	0.001

Abbreviations: ALT, alanine transaminase; CBF, cerebral blood flow; *pr*, partial correlation coefficient; L, left; R, right.
